# Supplementary material for: Effect of an Acute Resistance Training Bout and Long-Term Resistance Training Program on Arterial Stiffness: A Systematic Review and Meta-Analysis
Source: J Clin Med. 2021 Aug 7;10(16):3492. doi: 10.3390/jcm10163492 (PMC8397161; doi:10.3390/jcm10163492)
Supplement: Supplementary file 1 [file jcm-10-03492-s001.zip › jcm-1282660-supplementary.pdf]

Effect of an Acute Resistance Exercise Bout or Long-term Resistance Exercise Programs Alone and in Combination on Arterial Stiffness: A Systematic Review and Meta-Analyses

Roman Jurik [1,*], Aleksandra Zebrowska [2] and Petr Stastny [1,*]

[1] Faculty of Physical Education and Sport, Charles University in Prague
[2] Akademia Wychowania Fizycznego im. Jerzego Kukuczki w Katowicach
* Correspondence: stastny@ftvs.cuni.cz; Tel: +420 777198764

Corresponding author

Roman Jurik, PhD student

Charles University in Prague

Faculty of Physical Education and Sport

José Martího 269/31, 162 52 Prague, The Czech Republic

E-mail: rjurik@centrum.cz

Phone: +420 775 218 277

Table S1: Carotid – femoral pulse wave velocity before and after specific resistance training intervention.

Carotid - femoral pulse wave velocity (ms^{-1})										
Authors	Control group			Strength training			Strength and aerobic training			P values (afer intervention)
	Before	After	Change	Before	After	Change	Before	After	Change	
Cortez – Cooper et al. (2005)	7.24 (±0.83)	7.80 (±0.66)	-0.56 (±0.51)	7.91 (±0.88)	8.33 (±0.96)	-0.42 (±0.80)	N/A	N/A	N/A	P < 0.05
Casey et al. (2007)	6.9 (±0.2)	7.0 (±0.2)	-0.1 (±0.2)	6.5 (±0.1)	6.3 (±0.2)	0.2 (±0.15)	N/A	N/A	N/A	P > 0.47
Yoshizawa et al. (2009)	9.3 (±0.3)	9.5 (±0.3)	-0.2 (±0.3)	9.2 (±0.5)	9.0 (±0.5)	0.2 (±0.5)	N/A	N/A	N/A	P > 0.05
Guimaraes et al. (2010)[a]	10.23 (±1.82)	10.53 (±1.97)	-0.3 (±1.97)	10.15 (±1.66)	9.98 (±1.81)	0.17 (±1.81)	N/A	N/A	N/A	P ≥ 0.05
Beck et al. (2013)	7.02 (±0.17)	7.02 (±0.17)	0.01 (±0.17)	6.94 (±0.18)	6.81 (±0.18)	0.13 (±0.18)	N/A	N/A	N/A	P = 0.56
Croymans et al. (2015)	6.9 (±0.5)	7.2 (±0.2)	-0.45 (±0.31)	6.7 (±0.6)	6.6 (±0.8)	0.1 (±0.31)	N/A	N/A	N/A	P = 0.43
Au et al. (2017)[b]	5.9 (±0.7)	6.0 (±0.7)	-0.1 (±0.7)	6.24 (±0.56)	5.77 (±0.76)	0.47 (±0.7)	N/A	N/A	N/A	P < 0.05
Au et al. (2017)[c]	5.9 (±0.7)	6.0 (±0.7)	-0.1 (±0.7)	6.42 (±0.70)	5.72 (±0.60)	0.7 (±0.65)	N/A	N/A	N/A	P < 0.05

Data are expressed as mean (SD).

Abbreviations: N/A: Not available, [a] - continuous exercise training, [b] - heavier load, [c] – lighter load

Table S2: Femoral - ankle pulse wave velocity before and after specific resistance training intervention.

**Femoral - ankle pulse wave velocity (ms^{-1})**										
Authors	Control group			Strength training			Strength and aerobic training			P values (afer intervention)
	Before	After	Change	Before	After	Change	Before	After	Change	
Cortez – Cooper et al. (2005)	8.35 (±0.77)	8.48 (±0.64)	-0.13 (±0.63)	8.71 (±0.88)	8.62 (±1.06)	0.09 (±0.55)	N/A	N/A	N/A	$P < 0.05$
Yoshizawa et al. (2009)	7.55 (±0.2)	7.65 (±0.2)	-0.1 (±0.2)	7.5 (±0.2)	7.45 (±0.2)	0.05 (±0.2)	N/A	N/A	N/A	$P > 0.05$
Beck et al. (2013)	10.43 (±0.23)	10.3 (±0.23)	0.13 (±0.23)	10.41 (±0.25)	9.39 (±0.36)	1.02 (±0.31)	N/A	N/A	N/A	$P < 0.05$

Data are expressed as mean (SD).

Abbreviations: N/A: Not available

Table S3: Brachial - ankle pulse wave velocity before and after specific resistance training intervention.

Brachial - ankle pulse wave velocity (ms^{-1})										
Authors	Control group			Strength training			Strength and aerobic training			P values (afer intervention)
	Before	After	Change	Before	After	Change	Before	After	Change	
Okamoto et al. (2009)[a]	11.3 (±0.4)	11.2(±0.4)	0.1 (±0.4)	11.21 (±0.40)	12.66 (±0.54)	-1.54 (±0.47)	N/A	N/A	N/A	P < 0.05
Okamoto et al. (2009)[b]	11.3 (±0.4)	11.2(±0.4)	0.1 (±0.4)	11.55 (±0.35)	11.46 (±0.37)	0.09 (±0.36)	N/A	N/A	N/A	P < 0.05
Okamoto et al. (2009)[c]	10.7 (±0.4)	10.75 (±0.4)	-0.05 (±0.4)	10.49 (±0.37)	11.53 (±0.30)	-1.04 (±0.34)	N/A	N/A	N/A	P < 0.05
Okamoto et al. (2011)	10.2 (±1.5)	10.7 (±1.3)	-0.5 (1.4)	10.9 (±1.5)	10.2 (±1.3)	0.7 (±1.4)	N/A	N/A	N/A	P < 0.05
Figueroa et al. (2012)	10.8 (±0.3)	10.9 (±0.3)	-0.1 (±0.3)	11.1 (±0.3)	10.2 (±0.3)	0.9 (±0.3)	N/A	N/A	N/A	P < 0.05
Figueroa et al. (2013)	14.1 (±0.3)	14 (±0.4)	-0.1 (±0.4)	14.1 (±0.6)	12.8 (±0.4)	1.27 (±0.34)	N/A	N/A	N/A	P = 0.011
Lai et al. (2014)	16.2 (±4.4)	16.2 (±3.9)	0.01 (±1.7)	15.3 (±2.6)	14.6 (±2)	0.7 (±1)	N/A	N/A	N/A	P= 0.014
Miura et al (2015)[d]	16.4 (±2.1)	16.4 (±2)	0.016 (±0.8)	16.8 (±2)	15.5 (±2.1)	1.3 (±1.07)	N/A	N/A	N/A	P < 0.05

Data are expressed as mean (SD).

Abbreviations: N/A: Not available, [a] - upper limb training group, [b] - lower limb training group, [c] - group of resistance training with slow lifting and quick lowering, [d] - normotensive participants

Table S4: Effect size between intervention groups and controls in carotid - femoral PWV groups

Authors	Intervetion group	Control group	d value	95% C.I.	v value	S.E.
**High intensity resistance training programs (>80% 1RM)**						
Cortez – Cooper et al. (2005)	-0.42 (±0.80) n = 10	-0.56 (±0.51) n = 10	0.8644	0.4662	0.0413	0.258
Croymans et al. (2015)	0.1 (±0.31) n = 28	-0.45 (±0.31) n = 28				
Au et al. (2017)	0.47 (±0.7) n = 16	-0.1 (±0.7) n = 14				
**Authors**	**Intervetion group**	**Control group**	**d value**	**95% C.I.**	**v value**	**S.E.**
**Moderate intensity resistance training programs (60 - 80% 1RM)**						
Beck et al. (2013)	0.13 (±0.18) n = 15	0.01 (±0.17) n = 15	1.0624	0.6271	0.0493	0.023
Casey et al. (2007)	0.2 (±0.15) n = 24	-0.1 (±0.2) n = 16				
Yoshizawa et al. (2009)	0.2 (±0.5) n = 11	-0.2 (±0.3) n = 12				
**Authors**	**Intervetion group**	**Control group**	**d value**	**95% C.I.**	**v value**	**S.E.**
**Low intensity resistance training programs (<60 % 1RM)**						
Au et al. (2020)	0.7 (±0.65) n = 16	-0.1 (±0.7) n = 14	N/A	N/A	N/A	N/A

Data are expressed as mean (SD).
S.E. Standard error of the mean

Table S5: Effect size between intervention groups and controls in femoral – ankle PWV groups

Authors	Intervetion group	Control group	d value	95% C.I.	v value	S.E.
**High intensity resistance training programs (>80% 1RM)**						
Cortez – Cooper et al. (2005)	0.09 (±0.55)<br>n = 10	-0.13 (±0.63)<br>n = 10	N/A	N/A	N/A	N/A
**Authors**	**PRE values**	**POST values**	**d value**	**95% C.I.**	**v value**	**S.E.**
**Moderate intensity resistance training programs (60 - 80% 1RM)**						
Yoshizawa et al. (2009)	0.05 (±0.2)<br>n = 11	-0.1 (±0.2)<br>n = 12	1.3662	0.7682	0.0931	0.485
Beck et al. (2013)	1.02 (±0.31)<br>n = 15	0.13 (±0.23)<br>n = 15				

Data are expressed as mean (SD).
S.E. Standard error of the mean

Table S6: Effect size between intervention groups and controls in brachial – ankle PWV groups

Authors	Intervetion group	Control group	d value	95% C.I.	v value	S.E.
**Moderate intensity resistance training programs (60 - 80% 1RM)**						
Okamoto et al. (2009)[a]	-1.54 (±0.47)<br>n = 10	0.1 (±0.4)<br>n = 10	-1.4094	-1.9748	0.0832	0.482
Okamoto et al. (2009)[b]	0.09 (±0.36)<br>n = 10	0.1 (±0.4)<br>n = 10				
Okamoto et al. (2009)[c]	-1.04 (±0.34)<br>n = 10	-0.05 (±0.4)<br>n = 10				
**Authors**	**Intervetion group**	**Control group**	**d value**	**95% C.I.**	**v value**	**S.E.**
**Low intensity resistance training programs (<60 % 1RM)**						
Okamoto et al. (2011)	0.7 (±1.4)<br>n = 13	-0.5 (±1.4)<br>n = 13	1.0162	0.7074	0.0248	0.141
Figueroa et al. (2012)	0.9 (±0.3)<br>n = 5	-0.1 (±0.3)<br>n = 5				
Lai et al. (2014)	0.7 (±1)<br>n = 19	0.01 (±1.7)<br>n = 19				
Miura et al (2015)	1.3 (±1.07)<br>n = 53	0.016 (±0.8)<br>n = 55				

Data are expressed as mean (SD).
S.E. Standard error of the mean

N/A: Not available, [a] - upper limb training group, [b] - lower limb training group, [c] - group of resistance training with slow lifting and quick lowering

Table S7: Strengthening the Reporting of Observational Studies in Epidemiology (PEDro) checklist

Item	1	2	3	4	5	6	7	8	9	10	11
Cortez-Cooper et al. (2005)	Y	N	N	Y	N	N	N	Y	N	Y	Y
DeVan et al. (2005)	Y	Y	N	Y	N	N	N	Y	N	Y	Y
Casey et al. (2007)	Y	Y	Y	Y	N	N	N	Y	Y	Y	Y
Okamoto et al. (2009) [47]	Y	Y	Y	Y	N	N	N	Y	Y	Y	Y
Okamoto et al. (2009) [48]	Y	Y	Y	Y	Y	N	N	Y	Y	Y	Y
Yoshizawa et al. (2009)	Y	Y	Y	Y	N	N	N	Y	Y	Y	Y
Barnes et al. (2010)	Y	Y	Y	Y	N	N	N	Y	Y	Y	Y
Yoon et al. (2010)	Y	Y	Y	Y	N	N	N	Y	Y	Y	Y
Okamoto et al. (2011)	Y	Y	Y	Y	Y	N	N	Y	y	Y	Y
Figueroa et al. (2012)	Y	Y	Y	Y	N	N	N	Y	Y	Y	Y
Beck et al. (2013)	Y	Y	Y	Y	N	N	N	Y	Y	Y	Y
Croymans et al. (2015)	Y	Y	Y	Y	Y	N	N	Y	Y	Y	Y
Lai et al. (2014)	Y	Y	Y	Y	N	N	N	Y	Y	Y	Y
Okamoto et al. (2014)	Y	Y	Y	Y	Y	N	N	Y	Y	Y	Y
Lefferts et al. (2015)	Y	N	N	Y	N	N	N	Y	Y	Y	Y
Miura et al. (2015)	Y	Y	Y	Y	N	N	N	Y	Y	Y	Y
Nitzsche et al. (2016)	Y	N	N	Y	N	N	N	Y	N	Y	Y

Au et al. (2017)	Y	Y	Y	Y	N	N	N	Y	Y	Y	Y
Kingsley et al. (2017)	Y	Y	Y	Y	N	N	N	Y	Y	Y	Y
Tomschi et al. (2018)	Y	Y	Y	Y	N	N	N	Y	N	Y	Y
Parks et al. (2020)	Y	Y	Y	Y	N	N	N	Y	N	Y	Y
Rodríguez-Perez et al. (2020)	Y	Y	Y	Y	Y	N	N	Y	N	Y	Y

The PEDro scale is based on the Delphi list developed by Verhagen and colleagues at the Department of Epidemiology, University of Maastricht *(Verhagen AP et al (1998). The Delphi list: a criteria list for quality assessment of randomised clinical trials for conducting systematic reviews developed by Delphi consensus. Journal of Clinical Epidemiology, 51(12):1235-41).* The list is based on "expert consensus" not, for the most part, on empirical data. Two additional items not on the Delphi list (PEDro scale items 8 and 10) have been included in the PEDro scale. As more empirical data comes to hand it may become possible to "weight" scale items so that the PEDro score reflects the importance of individual scale items.

The purpose of the PEDro scale is to help the users of the PEDro database rapidly identify which of the known or suspected randomised clinical trials (ie RCTs or CCTs) archived on the PEDro database are likely to be internally valid (criteria 2-9), and could have sufficient statistical information to make their results interpretable (criteria 10-11). An additional criterion (criterion 1) that relates to the external validity (or “generalisability” or “applicability” of the trial) has been retained so that the Delphi list is complete, but this criterion will not be used to calculate the PEDro score reported on the PEDro web site.

The PEDro scale should not be used as a measure of the “validity” of a study’s conclusions. In particular, we caution users of the PEDro scale that studies which show significant treatment effects and which score highly on the PEDro scale do not necessarily provide evidence that the treatment is clinically useful. Additional considerations include whether the treatment effect was big enough to be clinically worthwhile, whether the positive effects of the treatment outweigh its negative effects, and the cost-effectiveness of the treatment. The scale should not be used to compare the "quality" of trials performed in different areas of therapy, primarily because it is not possible to satisfy all scale items in some areas of physiotherapy practice.

Notes on administration of the PEDro scale:

All criteria Points are only awarded when a criterion is clearly satisfied. If on a literal reading of the trial report it is possible that a criterion was not satisfied, a point should not be awarded for that criterion.

Criterion 1 This criterion is satisfied if the report describes the source of subjects and a list of criteria used to determine who was eligible to participate in the study. The search was limited by age. We included adults only with normotension. The search was not limited by gender.

Criterion 2 A study is considered to have used random allocation if the report states that allocation was random. The precise method of randomisation need not be specified. Procedures such as coin-tossing and dice-rolling should be considered random. Quasi-randomisation allocation procedures such as allocation by hospital record number or birth date, or alternation, do not satisfy this criterion.

Criterion 3 Concealed allocation means that the person who determined if a subject was eligible for inclusion in the trial was unaware, when this decision was made, of which group the subject would be allocated to. A point is awarded for this criteria, even if it is not stated that allocation was concealed, when the report states that allocation was by sealed opaque envelopes or that allocation involved contacting the holder of the allocation schedule who was "off-site".

Criterion 4 At a minimum, in studies of therapeutic interventions, the report must describe at least one measure of the severity of the condition being treated and at least one (different) key outcome measure at baseline. The rater must be satisfied that the groups' outcomes would not be expected to differ, on the basis of baseline differences in prognostic variables alone, by a clinically significant amount. This criterion is satisfied even if only baseline data of study completers are presented. Key outcomes are those outcomes which provide the primary measure of the effectiveness (or lack of effectiveness) of the therapy. In most studies, more than one variable is used as an outcome measure. We included the following outcome measures: carotid – femoral pulse wave velocity before and after specific strength training intervention, femoral – ankle pulse wave velocity before and after specific strength trainig intervention, brachial - ankle pulse wave velocity before and after specific strength training intervention.

Criterion 5-7 Blinding means the person in question (subject, therapist or assessor) did not know which group the subject had been allocated to. In addition, subjects and therapists are only considered to be "blind" if it could be expected that they would have been unable to distinguish between the treatments applied to different groups. In trials in which key outcomes are self-reported (eg, visual analogue scale, pain diary), the assessor is considered to be blind if the subject was blind.

Criterion 5 The researchers were blinded.

Criterion 6 The participants were blinded.

Criterion 7 Researchers and participants were blinded.

Blinding occurred only for the researchers and not for the participants, due to the fact that they need to know the specifics of the training unit and program.

Criterion 8 This criterion is only satisfied if the report explicitly states both the number of subjects initially allocated to groups and the number of subjects from whom key outcome measures were obtained. In trials in which outcomes are measured at several points in time, a key outcome must have been measured in more than 85% of subjects at one of those points in time.

Criterion 9 An intention to treat analysis means that, where subjects did not receive treatment (or the control condition) as allocated, and where measures of outcomes were available, the analysis was performed as if subjects received the treatment (or control condition) they were allocated to. This criterion is satisfied, even if there is no mention of analysis by intention to treat, if the report explicitly states that all subjects received treatment or control conditions as allocated.

Criterion 10 A between-group statistical comparison involves statistical comparison of one group with another. Depending on the design of the study, this may involve comparison of two or more treatments, or comparison of treatment with a control condition. The analysis may be a simple comparison of outcomes measured after the treatment was administered, or a comparison of the change in one group with the change in another (when a factorial analysis of variance has been used to analyse the data, the latter is often reported as a group × time interaction). The comparison may be in the form hypothesis testing (which provides a "p" value, describing the probability that the groups differed only by chance) or in the form of an estimate (for example, the mean or median difference, or a difference in proportions, or number needed to treat, or a relative risk or hazard ratio) and its confidence interval.

Criterion 11 A point measure is a measure of the size of the treatment effect. The treatment effect may be described as a difference in group outcomes, or as the outcome in (each of) all groups. Measures of variability include standard deviations, standard errors, confidence intervals, interquartile ranges (or other quantile ranges), and ranges. Point measures and/or measures of variability may be provided graphically (for example, SDs may be given as error bars in a Figure) as long as it is clear what is being graphed (for example, as long as it is clear whether error bars represent SDs or SEs). Where outcomes are categorical, this criterion is considered to have been met if the number of subjects in each category is given for each group.
